# Biotic pressure outweighs the influence of resource availability on physical defence investment in a tropical tree

**DOI:** 10.1093/aobpla/plaf027

**Published:** 2025-05-16

**Authors:** Bio Barriou Babah Daouda, M’Mouyohoun Kouagou, Eméline P S Assede, Orou G Gaoue

**Affiliations:** Faculty of Agronomy, University of Parakou, Parakou BP 123, Benin; Faculty of Agronomy, University of Parakou, Parakou BP 123, Benin; Faculty of Agronomy, University of Parakou, Parakou BP 123, Benin; Department of Plant and Soil Sciences, University of Pretoria, Pretoria, South Africa; Faculty of Agronomy, University of Parakou, Parakou BP 123, Benin; Department of Ecology and Evolutionary Biology, University of Tennessee, Knoxville, TN 37996, United States; African Centre for DNA Barcoding, Department of Botany and Plant Biotechnology, University of Johannesburg, APK Campus, Johannesburg, South Africa; Natural History and Conservation

**Keywords:** herbivory, *Acacia sieberiana*, browsing pressure, physical defence, elephant, piecewise structural equation models, soil resources, African savanna

## Abstract

Plants have long-lasting and complex interactions with herbivores, including insects and mammals. In response to high herbivory rates, plants either tolerate biomass loss or develop several defence mechanisms, such as physical defence. The resource availability hypothesis (RAH) predicts that plant defence investment is dependent on resource availability and plant's life history. However, the effect of resource availability on plant investment in defence is mediated through biotic pressure. We tested the effects of soil qualities and browsing pressure on the physical defence and reproductive investments in *Acacia sieberiana* at the Pendjari Biosphere Reserve in West Africa. We selected six populations, including three in the Pendjari River floodplain where soil moisture is high but with high elephant browsing pressure, and three populations on the plateau in the hunting zone where soils are drier and relatively poorer with a lower density of elephants. We found greater investment in physical defence for trees in the floodplain. Furthermore, *A. sieberiana* trees produced less fruit in the floodplain than in the plateau. Contrary to the predictions of the RAH, we found more and longer thorns in populations in the richer floodplains than on the plateau. This was linked to higher elephant browsing pressure in the floodplains. This physical defence was probably induced to cope with the episodic but high levels of herbivory observed in this environment. Surprisingly, the negative influence of thorn number and size on the likelihood of elephant damage was observed only in the rich floodplains and not in plateau sites. Altogether, our study demonstrates that the influence of resource availability (soil moisture, pH, and fertility) in shaping plant physical defence can be outweighed by high herbivory pressure.

## Teaser

Plants defend themselves from herbivores using features like thorns. According to the resource availability hypothesis, a plant’s defenses depend on soil quality and its life cycle. We studied Acacia sieberiana in West Africa's Pendjari Biosphere Reserve, comparing two areas: fertile floodplains with many elephants and drier plateaus with fewer elephants. Surprisingly, trees in the richer floodplains had more and longer thorns but fewer fruits. This suggests that high herbivore pressure, rather than just soil quality, influences plant defense. Interestingly, thorns helped reduce elephant damage only in the floodplain. Our study shows that herbivore pressure can be more important than soil quality in shaping plant defense strategies.

## Introduction

Herbivores have a major impact on plants and shape populations and communities (Edenius et al. 2011, Pringle et al. 2016, Wedel et al. 2021). Herbivore–plant relations can be affected by resource availability (Coley et al. 1985, Wise and Abrahamson 2007, Lin et al. 2021). The resource availability hypothesis (RAH) posits that species from high-resource environments grow fast and allocate little to herbivore-resistance traits, whereas those species in low-resource environments grow slowly and are highly resistant to herbivores (Hahn and Maron 2016). Because of the higher cost of leaf production, plants should invest more in their anti-herbivory defences in drier environments than in moist environments (Coley et al. 1985). It assumes that the rate of growth of a species depends on the amount of resources available in the environment and that there is an evolving trade-off between the allocation of resources to growth and defence. Finally, it also assumes that in a resource-poor environment, offsetting growth following defoliation is more difficult, and this selects species with higher investment in defensive strategies. Fast-growing species that can quickly allocate resources and species into rich habitats that can quickly mobilize resources for rapid growth can tolerate herbivory and, therefore, invest less in defence (Endara and Coley 2011). On the other hand, slow-growing species or species in resource-poor environments that cannot tolerate a significant and costly loss of biomass will invest more in direct defence. As a result, an increase in plant investment in defence is expected in resource-poor environments (Grime 1977, Southwood 1977, Chapin 1980). The RAH predicts that for slow-growing species, the opportunity cost of defence will be low and the negative impact of herbivory high. Therefore, slow growers should exhibit higher investments in constitutive defences (Coley 1987).

However, recently, the theoretical framework for the RAH has been nuanced (Hahn and Maron 2016). Recent studies suggest that while the theory of resource availability has empirical support across species, we have mixed evidence for intraspecific variation in defence investments (Hahn and Maron 2016, 2018, Hahn et al. 2019). Resource availability may have independent indirect effects on different components of plant defence; tolerance is affected by constraints set by resources on growth rates or physiological traits, whereas the effect of resources on resistance is mediated through variation in herbivore pressure that occurs across gradients (Hahn and Maron 2016). Biotic factors (e.g. browsing pressure) exert strong selective pressures on plants to shape plant defensive traits (Woods et al. 2012, Anderson et al. 2015). How plants invest in anti-herbivory defence can also be influenced by herbivory pressure. Theory predicts that when herbivory pressure is recurrent and high, plants will invest in constitutive defence (Zangerl and Rutledge 1996, Ito and Sakai 2009, Bixenmann et al. 2016). However, where herbivory is rare or episodic, it is optimal for plants to invest in induced defence that can be developed as required to limit biomass loss (Huntzinger et al. 2004). Induced physical defence is common, but the drivers of this functional response have received limited attention from ecologists (Midgley and Ward 1996, Barton 2016, Garcia et al. 2021, Ojha et al. 2022). Physical defence traits are generally inducible (Barton 2016). Plants may produce induced defences, such as longer thorns, to cope with higher levels of herbivory (Young et al. 2025).

In this study, we tested the effects of habitat differences on tree functional traits, thorn size, and number. We investigated the size-dependent investment in plant physical defence and tree damage by elephants. *Acacia sieberiana* suffers from heavy browsing by elephants in the Pendjari Biosphere Reserve in Benin (Djogbenou et al. 2021). The species is found in two different habitats (plateau and floodplain) of the reserve with different soil conditions and varying elephant densities. Consistent with the hypothesis of an evolutionary trade-off between resource allocation to growth and defence from the resource availability theory, we hypothesized that (i) *A. sieberiana* plant size-dependent investment in physical defence (thorn number and length) would be greater in the resource-poor habitats in the plateau where soils are drier and less nutrient-rich than in the floodplain. Furthermore, (ii) a higher density of elephants in the floodplain would result in a higher rate of herbivory pressure, with a higher prevalence of broken branches than on the plateau. (iii) Finally, we hypothesized that greater elephant damage would reduce fecundity due to a greater allocation of resources to defence rather than fecundity.

## Materials and methods

### Study system

We studied the investment in physical defence for *A. sieberiana* in the Pendjari Biosphere Reserve (10° 30′-11° 30′ N and 00° 50′-2° 00′ E) in the Sudanian region of the Dahomey Gap at the northwestern tip of Benin (Assédé et al. 2012, Houehanou et al. 2017). The Biosphere reserve covers a total area of 575 000 ha and includes three zones: the Pendjari National Park (275 000 ha), the Pendjari Hunting Zone (175 000 ha), and the Atacora Hunting Zone (125 000 ha; PAPE-CENAGREF 2014). The climate of the Pendjari Biosphere Reserve is characterized by a rainy season (April/May–October) and a dry season (November–March). Total annual rainfall averages 1000 mm, 60% of which falls between July and September. The average annual temperature is 27°C. The reserve has tropical ferruginous soils except on hills, which are dominated by rocky outcrops and clay soil, and loamy soil in flooded areas (Willaine and Volkoff 1967). The vegetation is dominated by savannahs (wooded, shrubby, and herbaceous savannahs) with tree islands, dry forests, and gallery forests along the rivers (Assédé et al. 2012). The Pendjari is the major river in the reserve, with a permanent water flow. The river shapes the distribution of wildlife, with higher density along the floodplain, which declines gradually away from the river. The Pendjari Biosphere Reserve is home to 50 species of mammals, including buffalo (*Syncerus caffer*), lion (*Panthera leo*), elephant (*Loxodonta africana*), and hippopotamus (*Hippopotamus amphibius*; Sinsin et al. 2002).

*Acacia sieberiana* is 1 of the 11 species of *Acacia* present in the Pendjari Biosphere Reserve and heavily browsed by elephants (Assédé et al. 2012, Houehanou et al. 2017). The species is a Sahelo-Sudanian to Sudano-Guinean species, which grows on clayey and humid soils, riverbanks, sandbanks, in watercourses, wooded savannahs, swampy savannahs, and degraded gallery forests (Akoègninou et al. 2006). *Acacia* develops a strong mutualism with several ant species, but this relationship is limited by annual fires in the dry savannah of West Africa (Frizzo et al. 2012, Djogbenou et al. 2021). *Acacia sieberiana* is a tree that is 3–25 m high, with 0.6–1.8 m in diameter and a 6 m trunk height on average. The tree has a rough, yellowish, flaking bark with small rectangular grey-brown scales. This species has a strong physical defence with the production of thorns in pairs at the branch nodes. Thorns have pointed tips and are 0.6–12 cm long. *Acacia sieberiana* is most likely pollinated by insects such as wasps, flies, and bees, similar to other species in the same genus (Fleming et al. 2007). At maturity, *A. sieberiana* flowers at the end of the dry season, and at the beginning of the dry season, the species produces seed pods (Orwa et al. 2018) with brown, elliptical, or round seeds that are 7–12 mm in diameter (Arbonnier 2000).

### Sampling sites with different environmental traits and elephant density

**Figure 1.**
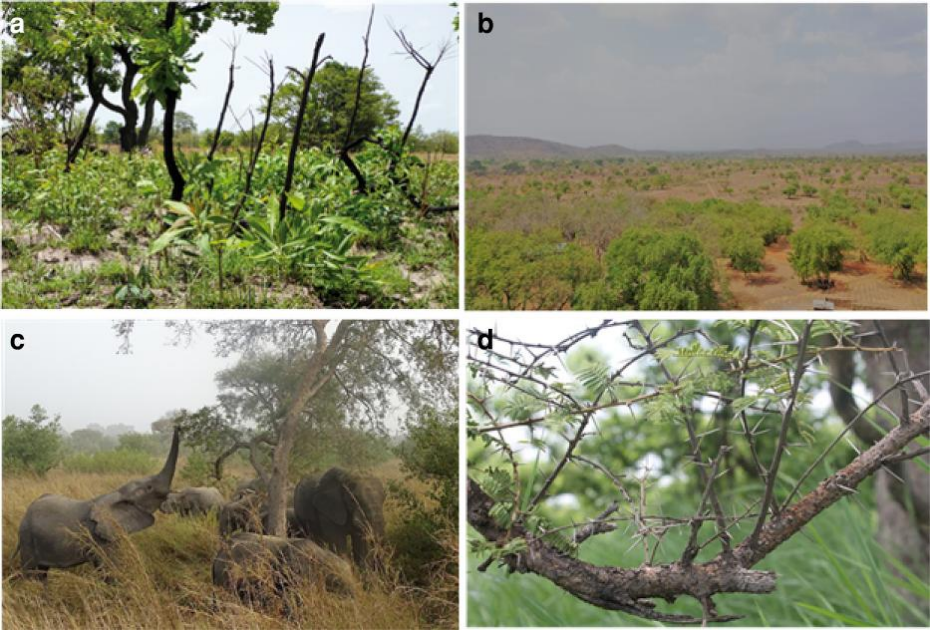
Comparative pictures of (a) the Pendjari River's floodplain with higher soil moisture and fertility and (b) the plateau, which has less fertile soils and lower soil moisture. Elephant and wildlife density varies between these two habitats, with the highest density in the floodplains where they exert a stronger (c) browsing pressure on *A. sieberiana* trees, which suffer major damages, and (d) develop numerous stronger and longer thorns as a physical defence. Source: Photo (a) E. Assédé; Photo (b) B. Babah Daouda; Photo (c) F. Amadou Bahleman; Photo (d) E. Assédé.

A previous study investigated the distribution and defensive mutualism in *Acacia* species in the study area (Djogbenou et al. 2021). This study revealed that *A. sieberiana* was the most common *Acacia* species in the Pendjari Biosphere Reserve and the species with the most elephant damage. We used this prior knowledge to locate and sample six populations of *A. sieberiana* equally distributed between the Pendjari National Park (hereafter floodplain) and the Pendjari Hunting Zone (hereafter plateau). The two areas have contrasting environmental characteristics (Agbossou and Okounde 2000) with different levels of resources (soil moisture, pH, and fertility). Floodplains are fertile, flooded by water that deposits layers of fine soil, rich mineral salts, nutrient-rich silt, and sediments over a large area. These sediments improve soil fertility with nutrients easily accessible to plants (Lima et al. 2002, Křížek et al. 2006, Kristen et al. 2008). We selected the first set of three populations in the Pendjari National Park located in the Pendjari River floodplain, where soil moisture (20%) was higher when compared with the remaining three populations established on the plateau in the Pendjari Hunting Zone, where soil moisture was 5% (Fig. 1). Soils in the floodplain were clayey silts with a pH between 6.5 and 7, and soils in the plateau were clayey sands with a pH of 6.5. Soil pH and moisture were assessed directly in the field. Soil moisture was measured using a handheld Extech MO750 soil moisture metre (Extech, Boston, MA, USA), and soil pH was measured using Hellige–Truog pH tester (Hellige, Inc., Long Island City, NY, USA).

The two sets of sites have different densities of elephants, which are the primary browsers of *A. sieberiana* (Djogbenou et al. 2021). These floodplains also have the highest wildlife density (Sinsin et al. 2002, Bouché et al. 2015, Houehanou et al. 2017, Rouamba and Hien 2022), which ensures varying *A. sieberiana* browsing pressure across the landscape (Djogbenou et al. 2021). The density of elephants was higher in the Pendjari National Park, which includes the floodplain (estimated at 0.04 individuals/km^2^) than in the hunting zone where the plateau sites are located, and elephant density was estimated at 0.005 individuals/km^2^ (PAPE-CENAGREF 2014). The total number of elephants was estimated at 1132 ± 427 in the Pendjari National Park and 48 ± 21 in the hunting zone (Rouamba and Hien 2022). Tree density and woody species diversity tend to have no effects on the spatial patterns of damage or severe damage caused by elephants (Calenge et al. 2002), which have a dietary preference for *Acacia* species (Douglas-Hamilton 1972, Calenge et al. 2002).

### Measuring tree functional traits, thorn size, and number

In each population, three transects of 50 m × 10 m were installed to collect demographic data from *A. sieberiana* individuals. In each transect, *A. sieberiana* individuals were tagged with aluminium tags. We sampled 103 individual plants in the floodplain and 115 in the plateau. For each *A. sieberiana* plant, we counted the number of fruits, measured the diameter at breast height (DBH) and total height, and estimated the intensity of elephant damage as the number of broken branches per tree (Jansson 2011). On each tree, we randomly selected three branches to count the number of thorns on the last 1 m of each branch (Young 1987). Ten thorns were randomly selected from each 1 m branch sample to measure their length and basal diameter using a digital calliper. The number of thorns and their width and length were used as metrics of physical defence in *A. sieberiana*.

### Statistical analysis

We first tested how *A. sieberiana* trees’ traits and physical investment varied between floodplain and plateau using generalized linear mixed-effect models (GLMMs). To test whether the number of fruits, number of thorns, and number of broken branches were significantly different between plateaus and floodplains, we developed GLMMs with negative binomial errors for the number of thorns and Poisson errors for the number of broken branches and fruits using *glmmTMB* package (Brooks et al. 2017) in R 4.3.1 (R Core Team 2024). We tested the same effects for thorn length, tree DBH, and height using a GLMM (Pinheiro and Bates 2000). We developed piecewise structural equation models (pSEMs) to estimate the direct and indirect effects of tree size on physical defence investment and reproductive effort using the *piecewiseSEM* package (Grace 2006, Lefcheck 2016). We developed separate pSEMs for floodplain and plateau to compare how underlying processes varied between habitats. In the pSEM, we first tested a meta-model that includes the number of thorns as predicted by tree DBH and total height, fruit number per tree as a function of thorn length, thorn number, and the number of broken branches in addition to DBH and height. This initial meta-model also included plant's height modelled as a function of DBH, and thorn length modelled as a function of tree DBH, height, and thorn number. For models that have a count response variable (number of fruits, number of thorns, and the number of broken branches), we used GLMMs with sites as random effect, using package *glmmTMB*. We used negative binomial errors for these models except for the number of broken branches for which there was overdispersion, and a Poisson model was appropriate. For the remaining two models with response variables as measurements (tree height and thorn length), we developed linear mixed-effect models with sites as random effect using the *lmer* function in package *lme4* (Bates et al. 2015).

We started with the same meta-model for both floodplain and plateau and conducted tests of direct separation to identify the missing paths to add to our model such that we obtain non-significant *P*-values (*P* > .05) for the model global goodness-of-fit test (Fisher's *C* or *lavaan* equivalent $\chi^{2}$). We obtained 0 degrees of freedom and non-significant *P*-values for the floodplain meta-model, indicating a good model fit (AIC = 22 698.26). For the plateau meta-model, we obtained significant *P*-values (Fisher's *C* = 40.42, *P* = 0, and $\chi^{2}$ = 31.35, *P* = 0, and AIC = 22 595.28). The test of directed separation indicates a missing path between thorn length and number. Including this missing path in the meta-model significantly improved the model by 29.35 units of AIC (AIC = 22 565.93 and *P* > .05). We retained this final meta-model. For the final meta-models for floodplain and plateau, we reported unstandardized path coefficients along with the marginal coefficient of variation (*R*^2^) for each endogenous variable.

## Results

### Effect of habitat differences on tree functional traits, thorn size, and number

**Figure 2.**
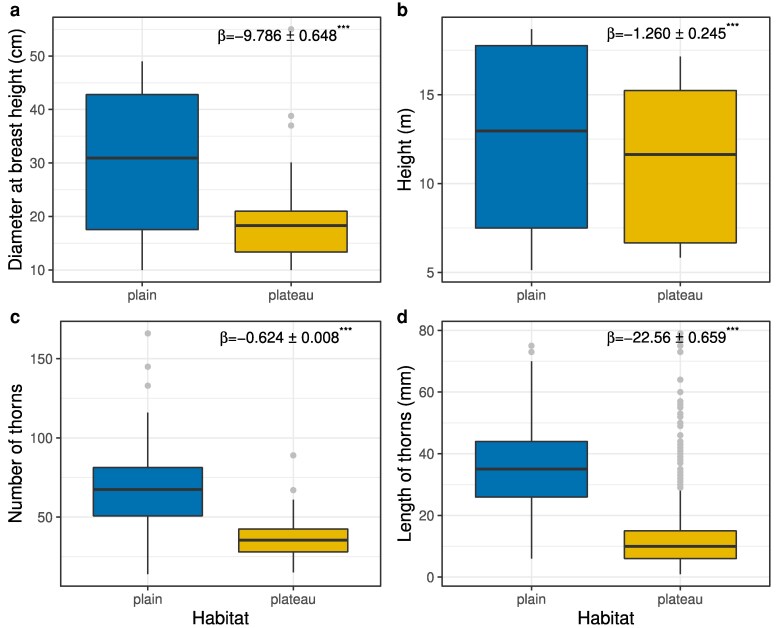
*Acacia sieberiana* trees (a) DBH (cm), (b) total height (m), (c) the number of thorns, and (d) thorn length (mm) were significantly greater in the floodplain than in the plateau sites. Sites in the floodplain are richer and have higher soil moisture than soils on the plateau, which are drier.

**Figure 3.**
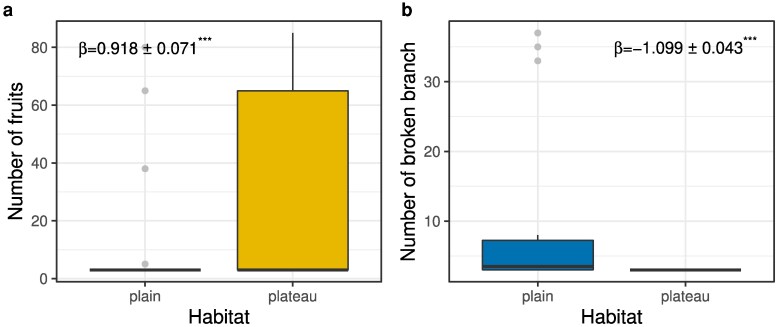
*Acacia sieberiana* trees had significantly fewer (a) fruits in the floodplain than in plateau sites but had more (b) broken branches by elephants in the floodplain than in sites on the plateau.

*Acacia sieberiana* trees were larger ($\hat{\beta }\;$ = −9.786 ± 0.648, *t* = −15.10, *P* < .001; Fig. 2a) and taller ($\hat{\beta }\;$ = −1.260 ± 0.245, *t* = −5.149, *P* < .001; Fig. 2b) in the floodplain than in the plateau. Similarly, the length and number of *A. sieberiana* thorns varied with soil conditions. *A. sieberiana* trees in the Pendjari floodplain had more thorns (median = 60 thorns) than in the plateau (median = 40 thorns) with greater dispersion of floodplain data ($\hat{\beta }\;$ = −0.624 ± 0.008, *t* = −79.32, *P* < .001; Fig. 2c). Trees in the Pendjari floodplains (median = 38 mm) also had longer thorns than those in the plateau (median = 25 mm), with a higher data dispersion in the floodplain populations ($\hat{\beta }\;$ = −22.56 ± 0.659, *t* = −34.24, *P* < .001; Fig. 2d). About 75% of trees in the floodplain had thorns that were more than 20 mm in length, while 75% of trees in the plateau had thorns that were <20 mm long (Fig. 3). Altogether, these results indicate greater investment in physical defence on soils with greater moisture content. Surprisingly, *A. sieberiana* trees produced fewer fruits in the floodplain than in the plateau ($\hat{\beta }\;$ = 0.918 ± 0.071, *t* = 13.02, *P* < .001; Fig. 3a). In addition, the number of broken branches due to elephants was higher in the floodplains than in the plateau ($\hat{\beta }\;$ = −1.10 ± 0.043, *t* = −25.49, *P* < .001; Fig. 3b), perhaps due to the much higher density of elephants in the floodplain.

### Size-dependent investment in plant physical defence and tree damage by elephants

**Figure 4.**
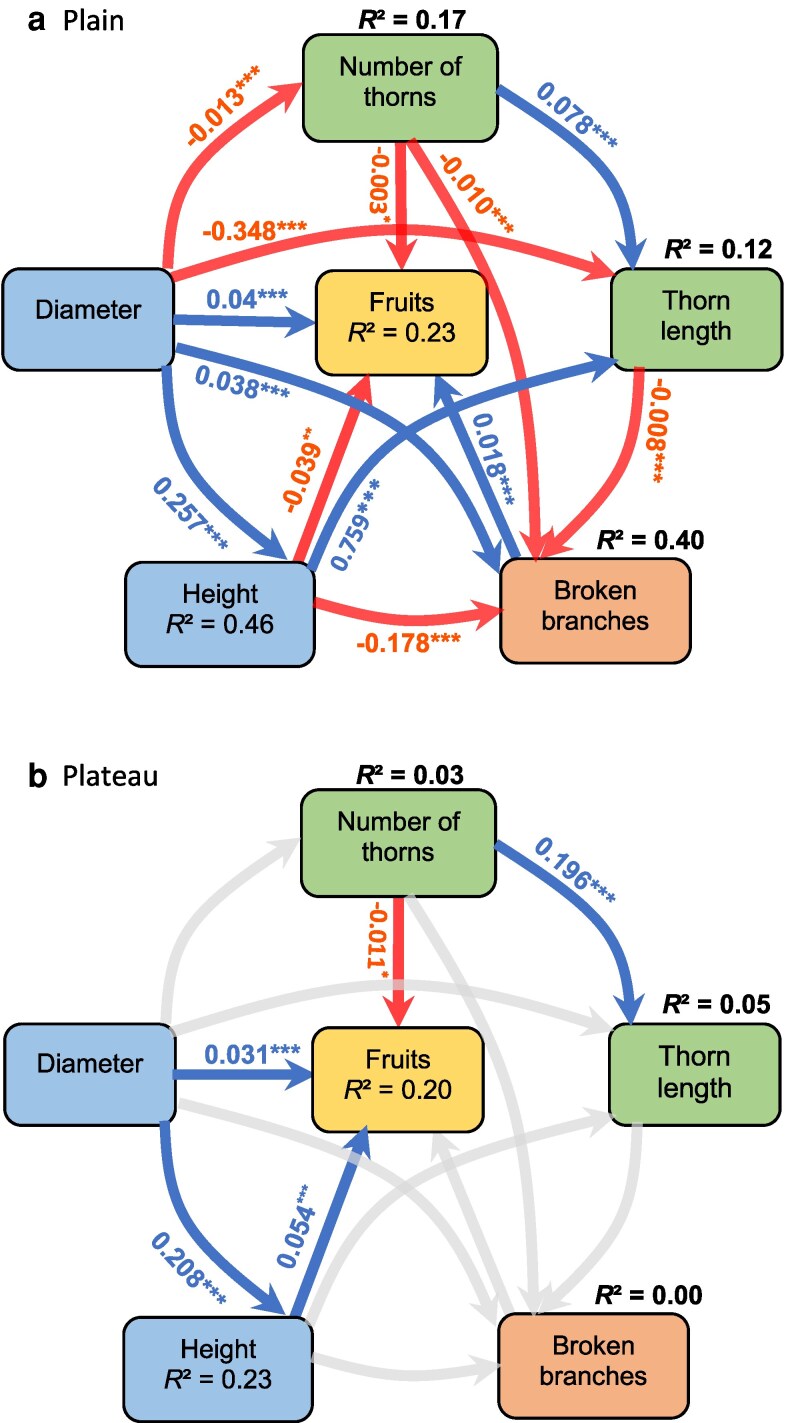
pSEM showing the direct and indirect relationship between *A. sieberiana* functional traits (tree DBH, total height), physical defence traits (thorn number, thorn length), the level of damage due to herbivory by elephants measured as the number of broken branches and tree reproductive investment (fruit number). These direct and indirect links were tested for natural populations of *A. sieberiana* in (a) the floodplain, which has richer and moist soils, and (b) the plateau, which is dry. The grey arrows indicate the statistically non-significant relationships, blue arrows indicate positive and statistically different relationships, and red arrows indicate negative and statistically different relationships. The degree of significance is indicated as *P < .05, **P < .01, ***P < .001.

**Figure 5.**
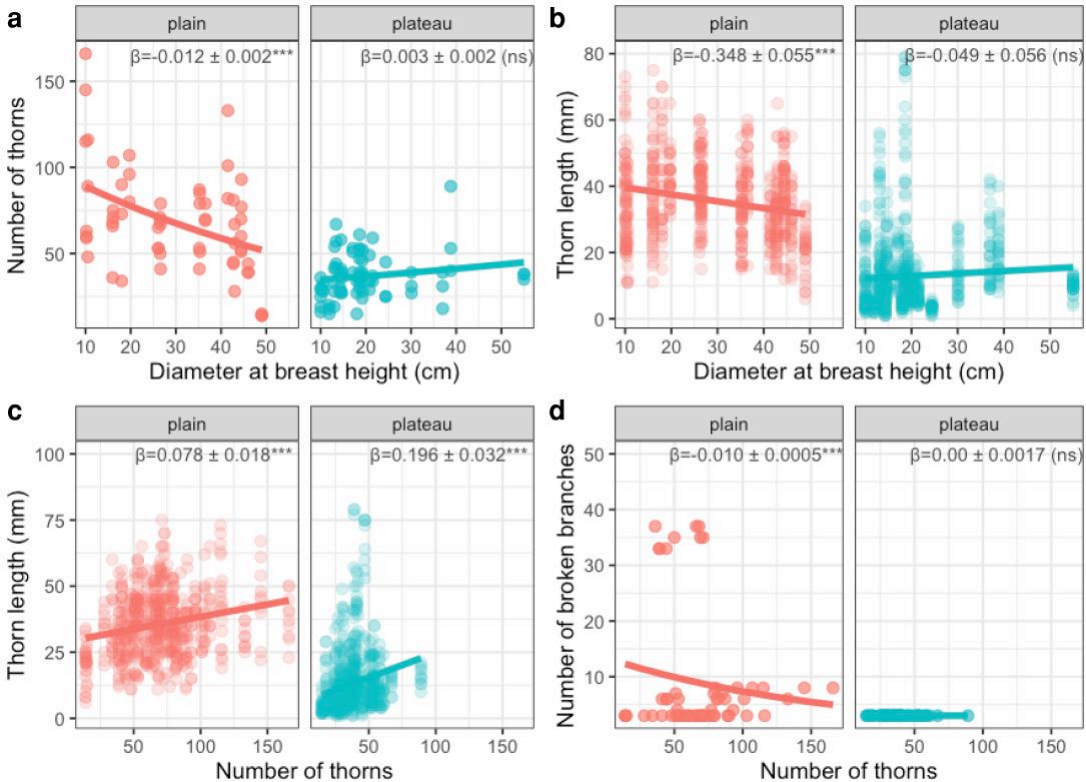
Effect of *A. sieberiana* tree DBH (cm) on (a) the number of thorns and (b) thorn length (mm) in floodplain and plateau. (c) Thorn number and length were positively associated in both habitats (in floodplain and plateau). (d) However, only in the floodplain was the number of broken branches negatively associated with the number of thorns per tree. For each relationship and for each habitat, *β* values indicate the slope of the relationship with the standard error of the mean. Asterisks represented the level of significance (***P < .001), and ‘ns’ indicated non-significant relationships.

**Figure 6.**
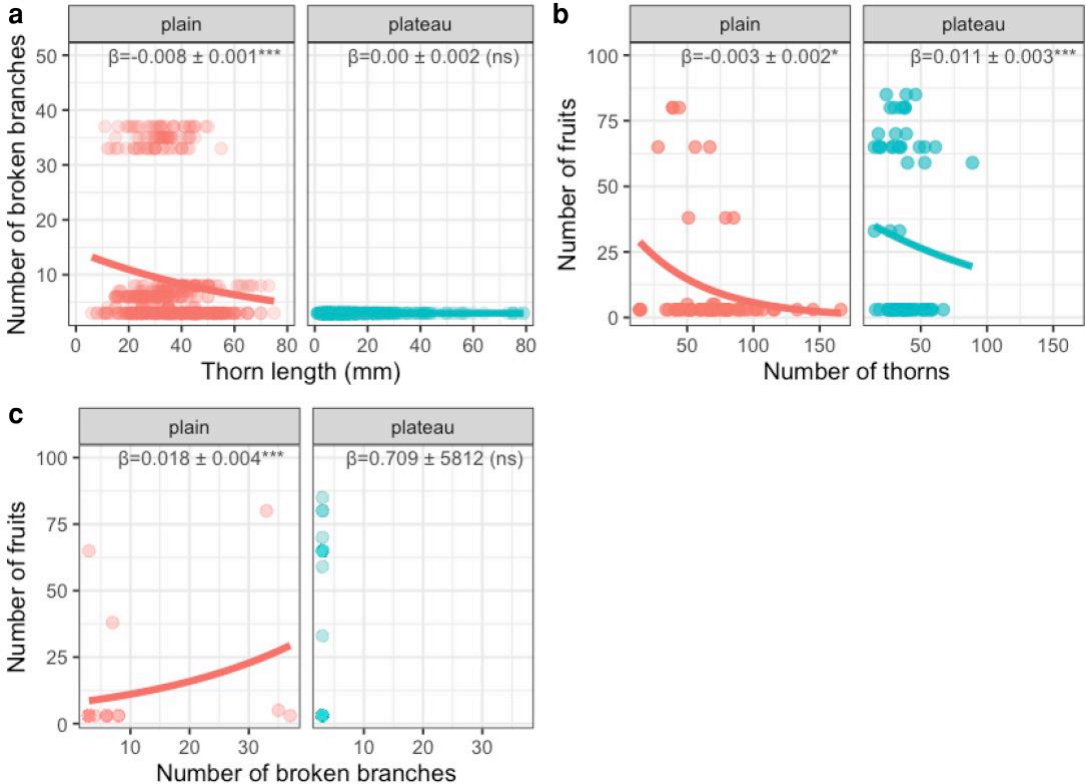
Effect of the length of thorns in *A. sieberiana* tree on (a) the number of broken branches, and effect of (b) the number of thorns and (c) number of broken branches on the number of fruits produced by tree in floodplain and plateau. For each relationship, and for each habitat, *β* values indicate the slope of the relationship with the standard error of mean. Asterisks represented the level of significance (***P < .001, *P < .05) and ‘ns’ indicated non-significant relationships.

The number ($\hat{\beta }\;$ = −0.012 ± 0.002, pSEM critical value *C* = −6.435, *P* < .001; Figs 4a and 5a) and length of *A. sieberiana* thorns decreased with tree DBH ($\hat{\beta }\;$ = −0.348 ± 0.055, *C* = −6.363, *P* < .001; Figs 4a and 5b) in the floodplain but not in the plateau ($\hat{\beta }\;$ = 0.003 ± 0.002, *C* = 1.866, *P* = .062 for thorn number, $\hat{\beta }\;$ = −0.049 ± 0.056, *C* = −0.890, *P* = .374 for thorn length, Figs 4b, 5a and b). Thorn length increased with tree height only in the floodplains ($\hat{\beta }\;$ = 0.759 ± 0.153, *C* = 4.960, *P* < .001; Fig. 4a) but not in the plateau (Fig. 4b). Thorn number and length were positively associated in the floodplain ($\hat{\beta }\;$ = 0.0776 ± 0.0179, *C* = 4.336, *P* < .001; Figs 4a and 5c) and in the plateau ($\hat{\beta }\;$ = 0.196 ± 0.032, *C* = 6.107, *P* < .001; Figs 4b and 5c). Trees with more thorns ($\hat{\beta }\;$ = −0.010 ± 0.0005, *C* = −18.460, *P* = .0001; Fig. 5d) or longer thorns ($\hat{\beta }\;$ = −0.008 ± 0.001, *C* = −6.611, *P* < .001; Fig. 6a) tended to have fewer broken branches in the floodplain (Fig. 4a) but not on the plateau (Fig. 4b). However, tree diameter was positively associated with the number of broken branches ($\hat{\beta }\;$ = 0.038 ± 0.002, *C* = 20.818, *P* < .001) but taller trees tended to have fewer broken branches ($\hat{\beta }\;$ = −0.178 ± 0.005, *C* = −34.517, *P* < .001) in the floodplain (Fig. 4a) but not on the plateau (Fig. 4b). Higher investment in thorn number was associated with fewer fruit produced both in the floodplain ($\hat{\beta }\;$ = −0.003 ± 0.002, *C* = −2.137, *P* = .033; Figs 4a and 6b) and plateau ($\hat{\beta }\;$ = −0.011 ± 0.003, *C* = −3.958, *P* = .0001; Figs 4b and 6b). In addition, trees with higher number of broken branches tended to produce more fruits in the floodplain ($\hat{\beta }\;$ = 0.018 ± 0.004, *C* = 4.851, *P* < .001; Fig. 4a) but not in the plateau (Figs 4b and 6c).

## Discussion

The RAH provides a theoretical framework that explains interspecific variation in plant defensive strategies (Endara and Coley 2011). This framework relates the evolution of defences to interspecific differences in inherent growth rate. In this study, we focused on intraspecific variation in plant defence across resource gradients to test whether the main predictions of the RAH apply within species. Studies have begun to explicitly examine the effects of herbivory pressure across resource gradients that might influence intraspecific patterns of plant growth and defence (Hahn and Maron 2016, Moreira et al. 2018, Croy et al. 2022). Incorporating the potential effects of biotic variables (herbivore pressure) on plant growth and defence traits should provide more robust inferences about the mechanisms regulating herbivore damage patterns and plant defence. In this study, we investigated the effect of habitat differences on the size-dependent investment in plant physical defence and tree damage by elephants.

### Effect of habitat differences on tree functional traits, thorn size, and number

Contrary to the predictions of resource availability theory (Coley et al. 1985, Endara and Coley 2011), we found longer and more thorns in *A. sieberiana* stand on the richer floodplains than on the plateau. Cost-effective defence is an important determinant of plant defence strategies (Coley et al. 1985). The theory of resource availability postulates that species adapted to resource-rich environments have higher growth rates and fewer constitutive defences but higher inducible defences. On the contrary, species adapted to low-resource availability environments will have lower growth rates and invest more in constitutive defence. In this study, our results show that *A. sieberiana* trees were taller and larger in the floodplains than on the plateau, suggesting faster growth rates in the plain. The difference in physical defence investment could be due to differences in browsing pressure between the two habitats rather than a plastic response of trees to different ecological conditions, consistent with previous studies on physical defence in *Acacia* (Young 1987, Zinn et al. 2007). Elephant damage rate was significantly higher in the floodplains than on the plateau, which is consistent with the higher density of elephants reported by previous studies in the floodplain (PAPE-CENAGREF 2014). Surprisingly, the negative influence of thorn number and size on the likelihood of elephant damage was observed only in the rich floodplains and not in plateau sites. These findings suggest that in the habitats with low herbivory pressure, physical defence investment may be less cost-effective, given the low return on investment in physical defence observed on the plateau. In addition, the influence of resource availability in shaping plant defence could be outweighed by high enough herbivory pressure. Clearly, the basis of the resource availability theory is higher tolerance to biomass loss and faster biomass regrowth post-herbivory in resource-rich habitats. That *A. sieberiana* was vigorously physically defended in the resource-rich floodplain points to weaker herbivory tolerance in this system, given the strong browsing pressure exert by the high density of elephants in the plains. In contrast, our study highlights the context dependence of physical defence investment consistent with the geographic mosaic theory of co-evolution (Thompson 1999). The Pendjari floodplains are flooded during the rainy season (PAPE-CENAGREF 2014), suggesting episodic herbivory, favourable to an investment in induced defence that can be developed as needed to limit biomass loss (Huntzinger et al. 2004). Physical defences are costly to express because of the large investment in structural carbohydrates needed to construct them (Bazely et al. 1991, Gómez and Zamora 2002, Kitajima et al. 2012). Thus, plants should benefit from having these traits be inducible to reduce construction costs until needed (Hoan et al. 2014).

### Size-dependent investment in plant physical defence and tree damage by elephants

We investigated the variation of physical defence in *A. sieberiana* and showed that the size-dependent investment in physical defence traits was a function of *A. sieberiana* tree height and diameter. The length of the *A. sieberiana* thorns decreased with tree diameter but increased with height. The number of thorns also decreased with tree diameter. In contrast, fruit production increased with diameter but decreased with tree height, suggesting a trade-off between size growth and reproduction. The reduced physical defence investment in larger trees is probably a consequence of higher investment in fruit production, given that fruit production was negatively associated with increasing thorn number but not thorn length (Minor and Kobe 2019). In contrast, higher investment of taller trees in thorn length was compensated with reduced fruit production.

*Acacia sieberiana* trees with more or longer thorns tended to have fewer broken branches. Similarly, previous studies in different systems showed that *A. sieberiana* trees which experienced high browsing intensity by giraffes had significantly longer thorns than trees with low browsing intensity (Ward and Young 2002, Zinn et al. 2007). High levels of herbivory can result in longer and denser thorns on branches exposed to herbivores (Milewski et al. 1991). For example, previous studies showed that thorns on shrubs in browsed sites were longer and sharper than in sites without browsing (Abrahamson 1975, Cooper and Owen-Smith 1986). The production of thorns in response to biomass damage by herbivores suggests an induced physical defence. In contrast to constitutive defence, induced defence is considered a cost-saving strategy in which defences are expressed only in response to herbivory (Bixenmann et al. 2016). The decrease in physical defence investment with tree diameter may also be due to a low herbivory pressure on trees with larger diameters that manage to resist browsing by small vertebrate herbivores. For example, short thorns are reported on the branches of large *Acacia drepanolobium* trees (Young 1987, Myers and Bazely 1991). In addition, long thorns were found on the lower branches of *A. drepanolobium* that are within reach of herbivores, and short thorns are found on the upper branches that are out of reach of herbivores (Young et al. 2003). Previous studies also showed that there is a near-absence of thorns on the branches that are not subjected to herbivory by mammals (Milewski et al. 1991). This is consistent with the higher investment in physical defence in taller *A. sieberiana* trees observed in our study system. Taller trees, unlike larger trees, which were more subject to elephant damage, were thus most likely to invest in higher constitutive defence.

Most studies tend to focus more on induced chemical defence in plants, and this has limited our understanding of the ecological and evolutionary underpinnings and consequences of induced physical defence (Barton 2016). However, several studies show that the induction of physical defence is equally as common as induced chemical defence (Midgley and Ward 1996, Ward and Young 2002, Barton 2016, Garcia et al. 2021, Ojha et al. 2022). However, the mechanism of inducibility for physical defence is less well understood than that of chemical defence (Barton 2016). The increase in thorn length as an induced defence can be confounded because the length of thorns can be allometrically related to branch thickness (Midgley and Ward 1996). Thorns are permanent investments, and their production is relatively slow (Young 1987, Armani et al. 2019). It takes several weeks for new thorns to reach their final size because the response is slow and irreversible. Thus, an induced response can only be sustained if herbivore pressure fluctuations are long-term phenomena; the presence or absence of herbivores must be constant. Controlled experiments will be necessary to better understand the temporal dynamics of the induction of physical traits. While detailed studies focused on identifying the timing of induction of secondary compound production, our understanding of the timing of induction for physical defence traits is limited (Fuchs and Bowers 2004). Because we controlled for the effect of individual size in our model, we suggest that the number of thorns produced could be a direct response to the browsing pressure of the elephants. Frequent browsing of top branches by elephants can limit branch growth and tree height. This could result in the development of short, thick annual branches with long thorns. In this case, we cannot exclude the possibility that the occurrence of long thorns could be a passive consequence of herbivory and not an actively induced defence (Milewski et al. 1991).

Research on the mechanisms underlying induced defence is particularly needed for woody plants in tropical regions (Barton 2016). Much of the early pioneering work, as well as recent work on the induction of spinescence, has been done on African *Acacia* trees (many of which have now been reorganized into different genera). These studies showed that plants exposed to browsing by large mammals, including gazelles, elephants, and giraffes, tend to have larger and greater densities of spines, thorns, and prickles than plants protected from browsers (Young 1987, Milewski et al. 1991, Gowda 1997, Young and Okello 1998, Gadd et al. 2001, Young et al. 2003, Scogings and Macanda 2005, Milewski and Madden 2006). In many of these studies, induced defence is inferred from the relaxation of spinescence following herbivore exclusion treatments in which densities or sizes of thorns and spines decrease following herbivore removal (Young and Okello 1998, Zinn et al. 2007). Although manipulative experiments in these systems are challenging, large mammals are the principal herbivores on these plants. Thus, a better understanding of the causes and consequences of their inducibility would shed light on the evolution of spinescence in the African flora (Barton 2016). Future research focusing explicitly on these ecological and evolutionary aspects of physical trait induction will be the most fruitful for moving this field closer to that of chemical induction, thereby greatly enhancing our understanding of herbivore-induced responses in plants.

## Data Availability

Data and R script used for the statistical analyses are publicly available and published on FigShare, an open-access data and code repository at https://doi.org/10.6084/m9.figshare.28743137.v1 (Gaoue and Babah Daouda 2025).
